# An *In vitro* Study of Bio-Control and Plant Growth Promotion Potential of Salicaceae Endophytes

**DOI:** 10.3389/fmicb.2017.00386

**Published:** 2017-03-13

**Authors:** Shyam L. Kandel, Andrea Firrincieli, Pierre M. Joubert, Patricia A. Okubara, Natalie D. Leston, Kendra M. McGeorge, Giuseppe S. Mugnozza, Antoine Harfouche, Soo-Hyung Kim, Sharon L. Doty

**Affiliations:** ^1^School of Environmental and Forest Sciences, College of the Environment, University of Washington Seattle, WA, USA; ^2^Department for Innovation in Biological, Agro-Food and Forest Systems, University of Tuscia Viterbo, Italy; ^3^Department of Biology, University of Washington Seattle, WA, USA; ^4^Wheat Health, Genetics and Quality Research Unit, USDA-ARS Pullman, WA, USA; ^5^Department of Plant Pathology, Washington State University Pullman, WA, USA

**Keywords:** bio-control, Salicaceae endophytes, soil borne plant pathogens, *Burkholderia*

## Abstract

Microbial communities in the endosphere of Salicaceae plants, poplar (*Populus trichocarpa*) and willow (*Salix sitchensis*), have been demonstrated to be important for plant growth promotion, protection from biotic and abiotic stresses, and degradation of toxic compounds. Our study aimed to investigate bio-control activities of Salicaceae endophytes against various soil borne plant pathogens including *Rhizoctonia solani* AG-8, *Fusarium culmorum, Gaeumannomyces graminis* var. *tritici*, and *Pythium ultimum*. Additionally, different plant growth promoting traits such as biological nitrogen fixation (BNF), indole-3-acetic acid (IAA) biosynthesis, phosphate solubilization, and siderophore production were assessed in all bio-control positive strains. *Burkholderia, Rahnella, Pseudomonas*, and *Curtobacterium* were major endophyte genera that showed bio-control activities in the *in-vitro* assays. The bio-control activities of *Burkholderia* strains were stronger across all tested plant pathogens as compared to other stains. Genomes of sequenced *Burkholderia* strains WP40 and WP42 were surveyed to identify the putative genes involved in the bio-control activities. The *ocf* and *hcnABC* gene clusters responsible for biosynthesis of the anti-fungal metabolites, occidiofungin and hydrogen cyanide, are present in the genomes of WP40 and WP42. Nearly all endophyte strains showing the bio-control activities produced IAA, solubilized tricalcium phosphate, and synthesized siderophores in the culture medium. Moreover, some strains reduced acetylene into ethylene in the acetylene reduction assay, a common assay used for BNF. Salicaceae endophytes could be useful for bio-control of various plant pathogens, and plant growth promotion possibly through the mechanisms of BNF, IAA production, and nutrient acquisition.

## Introduction

Biotic stress, especially due to pathogenic microorganisms, causes major crop losses worldwide which is equivalent to nearly $220 billion lost every year (Chakraborty and Newton, 2011). In response, growers often rely on a variety of chemicals to control these plant pathogens; however, such widespread use comes at both economic and environmental costs, causing undesirable consequences to human health through air, water, and soil pollution. Resistance to chemicals also is commonplace. Alternatively, the use of microbial organisms to manage plant diseases, termed bio-control, offers an environmentally-friendly and more sustainable replacement to the chemical pesticides. Several past studies demonstrated that endophytes have the potential to control many plant diseases caused by different plant pathogens (Ryan et al., 2008; Compant et al., 2010). Endophytic bacteria including *Aureobacterium, Bacillus, Paenibacillus, Phyllobacterium, Pseudomonas*, and *Burkholderia* recovered from host plants or seeds showed antagonistic activities against the plant pathogens, *Fusarium oxysporum, Rhizoctonia solani, Sclerotium rolfsii, Verticillium dahlia* and many other fungi (Chen et al., 1995; Rybakova et al., 2016).

Endophytes are bacterial and fungal communities that colonize the plant interior and contribute to the growth, development, fitness, and adaptation of the host plant (Rodriguez et al., 2008; Hardoim et al., 2015). Endophytes often confer considerable benefits to the host plants they inhabit. The diazotrophic (nitrogen fixing) endophytes can convert dinitrogen gas into nitrogen (N) compounds such as ammonium and nitrate, which are potentially available for N metabolism by the plant (Bhattacharjee et al., 2008; Santi et al., 2013). Furthermore, endophytes can also produce phytohormones and siderophores, and can solubilize inorganic phosphates (Khan et al., 2015; Santoyo et al., 2016). Siderophores are organic compounds that are produced by organisms during iron limiting conditions. Previous studies showed that plants can utilize microbial siderophores for iron acquisition. Iron deficient tomato plants supplemented with microbial siderophores, for example, produced higher crop yields, and had increased chlorophyll and iron content in the leaves (Radzki et al., 2013). In addition, siderophores are considered to be helpful in the biological control of plant pathogens (Verma et al., 2011; Ahmed and Holmström, 2014). Many plant associated rhizo- or endophytic bacteria can solubilize insoluble inorganic phosphates, which is potentially available for uptake by plants. Positive growth response has been reported in different crop plants inoculated with phosphate solubilizing endophytes (Manoel et al., 2015; Oteino et al., 2015; Passari et al., 2015). Endophytes also have the potential to synthesize phytohormones including IAA, gibberellic acid, cytokinin, and abscisic acid (Patten and Glick, 2002; Pirttilä et al., 2004; Feng et al., 2006; Sgroy et al., 2009; Shi et al., 2009; Videira et al., 2012).

Dozens of microbial endophyte strains were isolated from poplar and willow plants that may support the host plant growth in the nutrient limited, cobble-dominated riparian ecosystem in western Washington from which they were isolated (Doty et al., 2005, 2009). Several poplar and willow endophytes are diazotrophs with the ability of producing phytohormones and siderophores, and solubilizing inorganic phosphates (Khan et al., 2015; Doty et al., 2016). A recent study showed that inoculated Salicaceae endophytes in hybrid poplar plants can contribute about 65% of the total N in the leaves and increase plant biomass through BNF (Knoth et al., 2014). Additionally, a significant amount of IAA production has been observed by poplar endophytes *in vitro* (Xin et al., 2009a,b). Cross inoculation of poplar and willow endophytes in other plant species (rice, maize, tomato, pepper, grasses, and conifer seedlings) showed substantial growth enhancement in nutrient poor conditions (Khan et al., 2012, 2015; Kandel et al., 2015). Furthermore, inoculated maize plants with Salicaceae endophytes showed improvement in photosynthetic capacity (higher CO_2_ assimilation rate) of leaves and higher biomass, and also resulted in early flowering in tomato and pepper (Khan et al., 2012; Knoth et al., 2013).

*Burkholderia, Pseudomonas, Curtobacterium*, and *Sphingomonas* were the most common endophyte genera discovered in poplar and willow plants through culture dependent and independent methods (Doty et al., 2009, 2016). Previous studies have shown that plant associated endophytic or rhizospheric *Burkholderia* species can degrade toxic compounds, promote plant growth, fix atmospheric N, and inhibit the growth of plant pathogenic fungi or oomycetes (Perin et al., 2006; Suárez-Moreno et al., 2012; Mitter et al., 2013; Bernabeu et al., 2015). Furthermore, bio-control strains of bacteria release different metabolic compounds including antibiotics, and lytic enzymes effective in growth inhibition of phytopathogenic fungi or oomycetes (Compant et al., 2005; Gagne-Bourgue et al., 2013; Pageni et al., 2014). More recent studies suggested the potential application of *Burkholderia* in agriculture for plant growth promotion, and biological disease control (Govindarajan et al., 2008; Mattos et al., 2008; Paungfoo-Lonhienne et al., 2014; Bernabeu et al., 2015).

The objectives of this study were to explore the capabilities of Salicaceae endophytes to control the *in vitro* growth of several soil borne plant pathogens including *R. solani* AG-8, *Fusarium culmorum, Gaeumannomyces graminis* var. *tritici*, and *Pythium ultimum*. These are widespread pathogens of many economically important crops including small grain crops such as wheat and barley, grain legumes, and brassicas worldwide (Paulitz, 2006; Hane et al., 2014). Additionally, we aimed to discover the possible molecular mechanisms used by endophytes to arrest the fungal growth through genomic comparisons with known bacterial strains that produce anti-fungal metabolites. In order to more fully characterize the isolates, we tested the anti-fungal strains for other potential plant growth promoting abilities.

## Materials and methods

### Endophyte strains

Endophyte strains (except PD1) were isolated from poplar and willow plants that were collected at the Three Forks Natural Area in King County, WA in the riparian zone of the Snoqualmie River (47° 31′ 14.30″ N, 121° 46′ 28.32″ W). The plant samples were surface-sterilized with 10% bleach (10 min) and 1% Iodophor (5 min), and rinsed three times in sterile deionized water. Isolated colonies of individual endophyte strains from surface sterilized plant tissues were flash frozen in 33% (v/v) glycerol, and retained at −70°C for utilization in various studies. Many of the endophyte strains available in our collection, a total of 55 poplar and 4 willow strains (Table 1 and Table S1), were examined for their *in vitro* antagonistic activities initially against *R. solani* AG-8. The endophyte strains capable of *in vitro* growth inhibition of *R. solani* AG-8 were further tested against other plant pathogens including *F. culmorum, G. graminis* var. *tritici*, and *P. ultimum*. Thirteen out of the total number of strains had shown antagonistic activities against at least one plant pathogen. Two endophyte strains (PD1 and WW7) were characterized in previous studies (Doty et al., 2009; Khan et al., 2014), and the remaining 11 strains were characterized in this study. In addition, endophyte strains having antagonistic activities were further studied for different plant growth promoting traits including phosphate solubilization, siderophore production, BNF, and IAA production.

**Table 1:** Endophyte strains used in this experiment.

**Strain**	**Identity**	**Origin**	**Appearance**	**PGPA^a^**	**GenBank accession no**.	**rRNA sequence size, partial (bp)**
WP 4-2-2	*Burkholderia* sp.	Poplar root	Buff	IAA, NA	KU495920	903
WP 4-3-1	*Rhodotorula graminis*	Poplar stem	Pink	CLP, IAA	KU500895	554
WP 4-3-2	*Burkholderia* sp.	Poplar stem	Buff, mucoid	Sd, NA	KU500893	947
WP 4-3-3	*Curtobacterium* sp.	Poplar stem	Bright yellow	IAA	KU550576	962
WP 4-4-2	*Rahnella* sp.	Poplar stem	Buff, mucoid	IAA, Sd	KU500894	966
WP 4-4-6	*Pseudomonas* sp.	Poplar stem	Pale yellow	IAA, NA	KU500891	1,032
WP 4-5-3	*Rahnella* sp.	Poplar stem	Buff, mucoid	Sd, IAA	KU500892	1,076
WP 4-10-4	*Curtobacterium* sp.	Poplar leaf	Bright yellow	NA, IAA	KU550577	859
WP40	*Burkholderia* sp.	Poplar stem	Buff	*ofn, orb, hcn*, Sd, NA, IAA	KF597274	1,494
WP41	*Burkholderia* sp.	Poplar stem	Buff	*ofn, orb*, Sd, NA, IAA	KF597275	1,494
WP42	*Burkholderia* sp.	Poplar stem	Buff	*ofn, orb*, Sd, *hcn*, NA, IAA	KF597276	1,494
WW7	*Curtobacterium* sp.	Willow stem	Pale yellow	IAA, Sd, NA	KU523564	926
PD1	*Pseudomonas putida*	Poplar stem	Buff	IAA	KF443801	1,496
*Pf* 2-79	*P. fluorescens*	Soil	Bright yellow	CLP, *phz*		
*Pf* Q8r1-96	*P. brassicacearum*	Soil	Buff	CLP, *dapg, plt, prn*		

a *Plant growth promoting activities: cyclic lipopetide (CLP) determined using the drop collapse assay; indole acetic acid (IAA) determined on YEM medium plus L-trpytophan using Salkowski reagent; gene clusters for the antifungal metabolites occidiofungin (ofn), ornibactin (orb), hydrogen cyanide (hcn), diacetylphloroglucinol (dapg), phenazine (phz), pyoluteorin (plt) and pyrrolnitrin (prn) loci determined using PCR; siderophore (Sd) determined by orange halo on CAS medium, nitrogenase activity (NA) determined by acetylene reduction assay*.

### Fungal isolates, bacterial controls, and media

*Rhizoctonia solani* AG-8 isolate C1 (Weller et al., 1986) and *F. culmorum* isolate 70110023 from oat grain were cultured on potato dextrose (PD) agar. *Gaeumannomyces graminis* var. *tritici (Ggt)*, isolate ARS-A1 (Kwak et al., 2009) was maintained on 1/5X PD agar. *P. ultimum* isolate 217, isolated from a chickpea field at Spillman Farm, Pullman, Washington in 2014, was cultured on SY agar (5.0 g sucrose, 0.5 g Difco yeast extract per liter). To optimize growth for inhibition assays, each of the four fungi were transferred to Mannitol Glutamate/Luria (MG/L; Cangelosi et al., 1991), PD, 1/5X PD and SY agar, depending upon the isolate, and grown at 25°C in darkness for 5–10 d. Bacterial controls for inhibition assays were *Pseudomonas fluorescens* strain 2–79 (Thomashow et al., 1990), *P. protegens* Pf-5 (Howell and Stipanovic, 1980), and *P. brassicacearum* strain Q8r1-96 (Raaijmakers et al., 1999). These were cultured in Luria broth (LB) or MG/L at 27°C, 200 rpm for 16–18 h.

### Endophyte growth on fungal media

Endophytes were tested to determine whether endophyte growth was altered, especially inhibited, on the media used to culture fungi. Endophyte strains were grown in MG/L broth at 27°C, 200 rpm for 16–18 h. Cultures were diluted to an OD_630_ of 0.2 using MG/L, PD, 1/5X (1/5th strength) PD or SY broth. Four replicated volumes of 2.5 μL each were transferred to the different agar media. Diameters of the colonies were measured after 22, 40, 54, and 70 h of growth at 27°C.

### Dual plate inhibition assays

Each endophyte strain was cultured in either PD or MG/L broth, and diluted to an OD_630_ of 0.2 as above. Fungal pathogens were transferred to appropriate agar medium and grown at 25°C until the leading edge of the colony was 1–3 cm from the plate perimeter. For the assays, 5-mm agar disks were cored from the leading edge. The “dual plate” assay consisted of the fungus disk in the center of the plate and two 2.5 μL volumes of endophyte culture at opposite sides of the fungal disk, placed 1 cm from the perimeter of the plate. Assays were done on both PD and MG/L agar. Positive controls were *Pseudomonas* strains 2-79 and Q8r1-96 known to have bio-control activity; and negative bacterial controls were medium without bacteria.

Four plating regimens were developed to accommodate the differential between growth rates of the endophytes and each fungus: (1) Endophytes were plated about 80 h before *P. ultimum*, and inhibition was assessed 30–34 h later; (2) *R. solani* was plated 24 h before the endophytes, and inhibition was assessed 4.5 d later; (3) *Ggt* was plated about 46 h before the endophytes, and assessed for inhibition after 9 d; (4) *F. culmorum* was plated 3 d before the endophytes, with inhibition assessed after 9 d. Dual plate assays were incubated at 25°C in darkness. Experiments were done two to three times, with four replicates per strain per experiment.

Growth and inhibition measurements were taken when the fungus reached the outer edge of the control plate. Inhibition index was calculated as [y/(x + y)](100), where y is the distance between the center of the bacterial colony and edge of the fungus at the bacterial-fungal interface, and x is distance between the center of the agar plug and the leading edge of the fungus (McSpadden Gardener and Weller, 2001; Mavrodi et al., 2012). Only strains that were positive for antifungal activities were used to test further plant growth promoting properties, and investigated for potential molecular mechanisms through genome analyses in the cases where the genome had been sequenced.

### Drop collapse assay

Cyclic lipopeptide (CLP) surfactant production by the endophytes was assessed by transferring 5 μL of a 16–18-h non-diluted broth culture onto a hydrophobic surface, such as Parafilm (de Bruijn and Raaijmakers, 2009). Culture supernatants, obtained by centrifugation at 10,000 × g for 3 min, and 1:10 dilutions of supernatants were also tested. *Pseudomonas fluorescens* strains 2-79 and Q8r1-96 served as the positive and negative control, respectively. Three independent experiments of 3-5 replicates per strain were done.

### Inhibition of *R. solani* AG-8 by volatile compounds

Petri plates with center partitions (I plates, Carolina Biological Supply Company, Burlington, NC) were used to physically separate the source and target microbes. Assays for strains 4-4-2, 4-4-6, 4-5-3, 4-10-4, WP40, WP41, *Pseudomonas brassicacearum* Q8r1-96 and *P. protegens* Pf-5 were done on PDA; those for 4-3-2 and *P. fluorescens* 2-79 were done on MG/L agar. Strains WP41 and 2-79 were negative controls for the PDA and MG/L sets, respectively. No-endophyte controls (media only) for both PDA and MG/L also were included. An agar plug of *R. solani* AG-8 was transferred to the perimeter of one half of the I plate and incubated at 25°C for 24 h. Endophytes were cultured in MG/L broth as described previously, and cultures were diluted to OD_630_ of 0.2. A 2.5-μL volume of diluted culture was applied to the center of the second half of the I plate 24 h after the pathogen. Plates were wrapped with Parafilm and incubated for an additional 3–4 d. When the leading edges of the *R. solani* colony in the negative controls were close to, but not touching, the center partition, pathogen colony radii were measured in all plates. Each endophyte was tested in triplicate for each of two experiments.

### Phosphate solubilization assay

Phosphate solubilizing properties of poplar and willow endophytes were determined using National Botanical Research Institute's Phosphate (NBRIP) agar medium (Nautiyal, 1999). A few colonies of individual endophyte strains were introduced per NBRIP plate in quadruplicate positions using sterile inoculating sticks (Puritan Medical Products Company, Maine). The clear halo area around an endophyte colony was observed after incubating 1 week at 30°C. The phosphate solubilization index (PSI) was calculated in endophyte strains capable of producing a distinct clear halo as a zone of solubilization (Khan et al., 2015). The poplar endophyte WP5 (Doty et al., 2009) was used as a reference strain for comparisons.

### Siderophore production assay

M9 minimal medium supplemented with Chrome Azurol S (CAS) was used for the siderophore production assay. Minimal medium (Yun et al., 2000) was prepared, autoclaved, and mixed with filter-sterilized, pre-warmed MgSO_4_ (2 mM final), CaCl_2_ (0.1 mM final), sucrose (0.2% final), and Casamino acids (0.9% final) (Loewen, 1984). CAS solution was prepared according to the protocol developed by Schwyn and Neilands (1987), autoclaved, and combined with the minimal medium prior to the assay. The area of color conversion from blue to orange around an endophyte colony was measured after incubating 1 week at 30°C for each endophyte strains per plate.

### IAA quantification assay

For IAA quantification, endophytes were grown in triplicate in 25 ml TYC broth (g L^−1^: 5 tryptone, 3 yeast extract, and 0.872 g CaCl_2_.2H_2_O) with 0.1% (w/v) L- tryptophan for 4 days in 125-ml flasks, and pelleted through centrifugation. One ml of the supernatant was incubated with 2 ml of Salkowski reagent (2 ml of 0.5 M FeCl_3_, and 98 ml of 35% HClO_4_; Gordon and Weber, 1951) for half an hour, and the optical density at a wavelength of 530 nm was observed (Xin et al., 2009a). A standard curve was developed with known amounts of IAA using Salkowski reagent and TYC broth with tryptophan and without endophytes, and the IAA amount produced by each endophyte strain was calculated using the standard curve.

### Acetylene reduction assay (ARA)

Endophyte strains were grown overnight at 30°C in MG/L or N-limited combined carbon medium (NLCCM) on a rotatory shaker. The overnight grown strains in MG/L cultures were microfuged at 4°C at 8,000 rpm for 10 min, combined with NLCCM cultures already in progress, and incubated on a shaker for another night. Cell density was determined through spectrophotometry, and the OD_600_ was adjusted to 1.0. Sixteen milliliter culture of each strain was transferred into balch tubes, dosed with 500 μl acetylene, and incubated for 24 h. Three tubes per strain were prepared; two were for the dosed cultures and one was for undosed control. After 24 h, 5 ml air from autosampler vials was removed and replaced with 5 ml headspace from the balch tubes. Samples were analyzed in the DeLuca Biogeochemistry Lab at the University of Washington, Seattle.

### Analysis of the nitrogenase subunit gene, *nifH*

The nitrogenase subunit gene, *nifH*, was amplified through PCR using universal *nifH, nifH*-b1 (Bürgmann et al., 2004), and *nifH* (Poly et al., 2001) primers. Colony PCR was performed in the same way as described below.

### Identification of endophytes through 16S/26S rRNA sequencing

Overnight grown bacterial colonies were used for colony PCR by transferring a single colony into 20 μl of sterile water, vortexing briefly, and using 1 μl as DNA templates for PCR analysis. The universal primers for 16S rRNA gene, 8F (5′-AGAGTTTGATCCTGGCTCAG-3′) and 1492R (5′-GGTTACCTTGTTACGACTT-3′) were used to amplify the 16S rRNA, which produced 1.5 kb amplicon products. PCR reactions of 25 μl consisted of 1 μl template DNA, 12.5 μl of PCR premix Buffer E (EpiCentre, Madison, WI), 0.8 μl of each primer (at 0.2 μg μl^−1^), 0.3 μl of Taq DNA polymerase (New England BioLabs, Inc., Ipswich, MA), and 9.6 μl of sterile water. Amplified PCR products were incubated with ExoSAP PCR cleanup reagent (Affymetrix, Inc., Cleveland, OH) at 37°C for 30 min followed by 80°C for 15 min in P100 Thermal Cycler (Bio-Rad, Inc., Hercules, CA). The ExoSAP cleaned 16S rRNA PCR products were sequenced by the Sanger sequencing approach (GENEWIZ, South Plainfield, NJ). For the yeast strain WP 4-3-1, the D1/D2 region of the large subunit (26S) of rRNA was amplified using primers F63 (5′-GCATATCAATAAGCGGAGGAAAAG-3′) and LR3 (5′-CGTCCGTGTTTCAAGACGG-3′), and PCR products were sequenced as mentioned above.

The 16S/26S rRNA sequences of all poplar endophytes were deposited in GenBank (NCBI) database under accession numbers KF597274, KF597275, KF597276, KU495920, KU500894, KU500893, KU550576, KU500895, KU500892, KU500891, and KU550577 for WP40, WP41, WP42, WP 4-2-2, WP 4-3-1, WP 4-3-2, WP 4-3-3, WP 4-4-2, WP 4-5-3, WP 4-4-6, and WP 4-10-4 respectively.

### Comparative analysis of genomic regions related to the synthesis of anti-*Rhizoctonia* metabolites in *Burkholderia* species

The sequence of the entire genomes of *Burkholderia* spp. (WP40 and WP42) were compared against phylogenetically related strains where the synthesis of anti-fungal metabolites was functionally assessed through knock-out studies (Gu et al., 2009a). Other gene clusters encoding for putative non-ribosomal peptidase, polyketide synthases, and other enzymes involved in the synthesis of secondary metabolites characterized for a generic anti-microbial and anti-fungal effect were detected using antiSMASH, a bioinformatics tool for automatic genomic identification and analysis of biosynthetic gene clusters (Weber et al., 2015). We set a minimum ClusterFinder probability of 0.5 and searched for gene clusters with a minimum size of 4 open reading frames (orf) characterized by 5 or more biosynthesis PFAM-related domains. The presence of biosynthetic gene clusters for anti-fungal metabolites were assessed through a multi-genome alignment approach using Mauve (Darling et al., 2004). Gene clusters encoding for anti-fungal compounds used in this work as reference, are listed: occidiofungin (*ocf*) gene cluster from *Burkholderia contaminans* MS14 (EU938698.5); *afc* (AFC-BC11) gene cluster from *B. cepacia* BC11 (AF076477.1); pyrrolnitrin gene cluster *prn*ABCD from *B. pyrrocinia* CH-67 (AF161186.1); the polyketide synthases (PKSs) genomic island (locus tag: Bamb_5918-5933), and the 4-hydroxy-2-alkylquinolines (HAQs) biosynthetic gene luster hmqABCDEFG (locus tag: Bamb_5763-5769) from *B. cepacia* AMMD (GCA_000203915.1), *hcnABC* gene cluster form *P. fluorescens* PF5 (AF053760); the phenazine-1-carboxylic acid gene cluster phzABCDEFG from *P. fluorescens* 2-79 (L48616); the 2,4-diacetylphloroglucinol synthetic gene cluster phlABCDEF from *P. fluorescens* Q2-87 (U41818.1) and pyrrolnitrin gene cluster prnABCD from *P. fluorescens* (U74493).

### Assessing the plant growth promoting properties in *Burkholderia* spp. (WP40 and WP42) genomes

The draft genomes *Burkholderia* sp. WP40 and *Burkholderia* sp. WP42 had been sequenced and annotated at the Joint Genome Institute as a part of the sequencing project “Defining the functional diversity of the *Populus* root microbiome (Bioproject accession: PRJNA247585 and PRJNA247584).” Genomes are publically available at: https://img.jgi.doe.gov/. The Integrated Microbial Genome platform (Markowitz et al., 2012) was used for data-mining the genome of *Burkholderia* species WP40 and WP42 for genes with potential beneficial effects on plant fitness.

### Statistical analysis

Bartlett's test for homogeneity of the variances (Statistix vers. 8.1, Analytical Software, Tallahassee, FL) was applied to inhibition index values from replicated experiments. Mean values for inhibition index, percent inhibition (split plate assays) and endophyte colony diameter were obtained using ANOVA (Statistix vers. 8.1). Significant differences among means were determined using Fisher's protected least significant difference (LSD) test at *P* < 0.05.

One way analysis of variance, and Fisher pairwise comparisons were used for ARA and siderophore assays using Minitab 17 (Minitab Inc., State College, PA, USA). The single point measurement was presented in the IAA assay.

## Results

### Identification of endophytes

Poplar endophytes were identified through 16S/26S rRNA gene sequencing. The 16S/26S rRNA gene for each strain was sequenced and identified using BLAST on the NCBI database from the National Center for Biotechnology Information (www.ncbi.nlm.nih.gov/BLAST). As shown in Table 1, the best 16S rRNA matches of poplar endophytes were *Burkholderia, Rahnella, Curtobacterium*, and *Pseudomonas*.

### Endophyte growth on MG/L and PDA

To determine the optimal agar medium for inhibition assays, the endophytes were grown on MG/L, PDA, 1/5X PDA, and SY agar that were used to culture the fungi. The endophytes grew best on either MG/L or PDA, but not on 1/5X PDA or SY, as indicated by colony size after 70 h of growth (Table 2). Colonies of strain PD1 on MG/L reached the maximum diameter, that is, before undergoing confluent growth, by 54 h. *Pseudomonas* WP 4-4-6 and all of the *Burkholderia* spp. grew more rapidly on PDA, whereas *Rhodotorula graminis, Rahnella aquatilis, Curtobacterium* spp. and *P. putida* PD1 appeared to prefer MG/L. Strain PD1 also produced a diffusible pale yellow pigment on MG/L that was not observed on PDA (data not shown).

**Table 2:** **Growth of endophytes on four agar media**.

**Strain**	**Colony diameter (mm)^1^**			
	**MG/L**	**PDA**	**1/5X PDA**	**SY**
WP 4-2-2	5.5 g	6.6 cd	5.0 b	4.5 g
WP 4-3-1	7.4 d	4.9 f	4.1 c	5.4 f
WP 4-3-2	5.9 efg	6.1 de	5.2 b	4.8 fg
WP 4-3-3	6.9 de	4.6 fg	5.0 b	5.2 fg
WP 4-4-2	11.2 a	7.2 bc	6.0 a	8.4 b
WP 4-4-6	5.8 fg	7.1 bc	5.9 a	5.0 fg
WP 4-5-3	8.6 c	7.8 b	4.9 b	9.2 a
WP 4-10-4	7.2 d	4.9 g	5.9 a	6.4 e
WP40	7.5 d	9.2 a	6.0 a	7.5 cd
WP41	6.8 def	9.1 a	5.1 b	7.8 bc
WP42	5.2 g	7.9 b	5.1 b	6.9 de
WW7	9.5 bc	5.8 e	5.0 b	6.4 e
PD1^2^	10.4 ab^2^	7.8 b	n.g.	5.0 fg

1 *Measured after 70 h at 27°C; starting diameter = 4.25–4.75 mm. n.g., no growth. Letters indicate significance (P < 0.05) classes of means within each column, determined by Fisher's LSD*.

2 *PD1 on MG/L was measured at 54 h*.

### Inhibition index on dual plate assays

**Figure 1:**
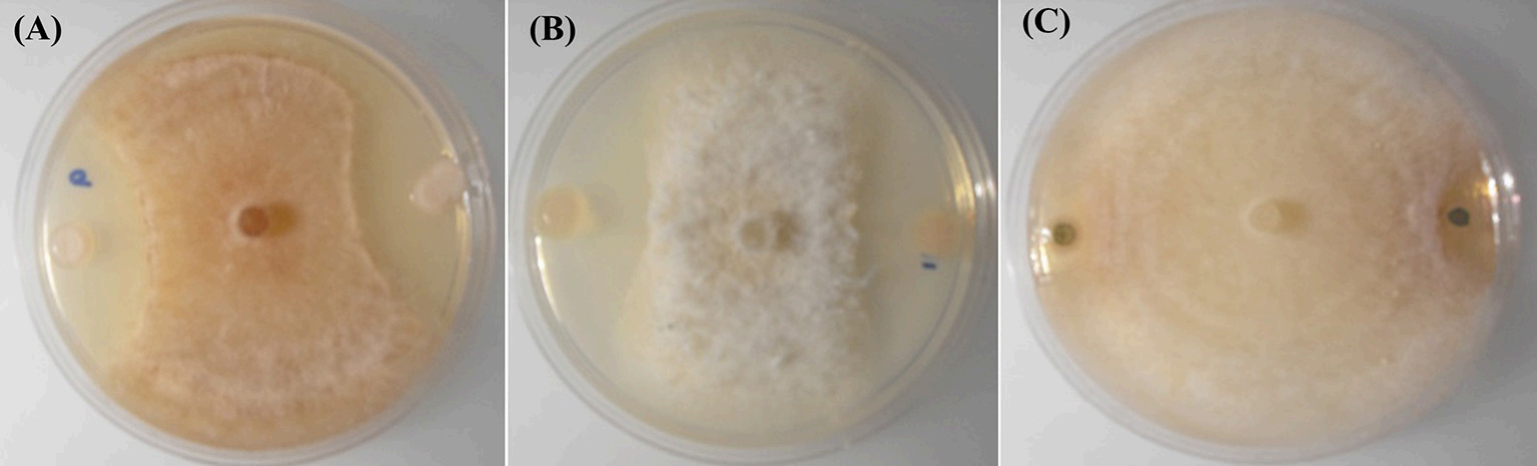
***In-vitro*** **inhibition of *R. solani* AG-8 (Plate A)**, and *Ggt* (Plate **B**) by poplar endophytes; WP40 and WP42 respectively; with reference to inhibition of *Ggt* by the positive control strain, *Pf* 2-79 (Plate **C**).

Dual plate inhibition assays were done using both MG/L and PDA, and significant differences in mean diameter of growth were determined using Fisher's least significant differences (Table 3). Of the 59 strains tested, 13 were positive for anti-fungal activity (Figure 1). The *Burkholderia* spp. WP40, WP41, WP42 consistently reduced fungal growth of all four pathogens on both media, and were as effective as the *Pseudomonas* controls 2-79 and Q8r1-96. *Burkholderia* 4-2-2 and *R. aquatilis* 4-4-2 displayed inhibition against three of the four pathogens only when grown on PDA. None of the endophytes were active against *F. culmorum* when grown on MG/L; however eight gave quantifiable inhibition on PDA. In contrast, PD1 showed inhibition against *Ggt, P. ultimum* and *R. solani* AG-8 when grown on MG/L but not on PDA. In general, *F. culmorum* and *Ggt* were responsive to more of the endophytes than were *P. ultimum* and *R. solani* AG-8. While endophyte strains 4-3-1, 4-3-3 and 4-10-4 had no activity against any of the pathogens on full-strength media, in our initial screening on ¼X PDA, all the endophyte strains listed in Table 1 showed some antagonistic activities against *R. solani* AG-8. Our findings suggest that nutrient composition has a role in the inhibitory activities of the endophytes. In some dual plate inhibition assays, we observed that *R. solani* AG-8 and *P. ultimum* did not grow to the perimeter of the agar plate if certain endophytes were present. The leading edge of the fungal hyphae in these cases was shorter than that of control plates (data not shown). The observations suggested the presence of inhibitory volatile compounds (VOCs).

**Table 3:** ***In vitro*** **inhibition of four soilborne fungal pathogens by bacterial strains on potato dextrose agar (PDA) and mannitol/glutamate agar (MG/L)**.

**Strain**	**Inhibition index^1^**						
	***F. culmorum***	***G. graminis tritici***		***R. solani*** **AG8**		***P. ultimum***	
	**PDA**	**PDA**	**MG/L**	**PDA**	**MG/L^2^**	**PDA**	**MG/L**
WP 4-2-2	10.0 c	29.0 a	na	na	na	8.0 b	na
WP 4-3-1	na	na	na	na^3^	na	na	na
WP 4-3-2	9.4 c	18.9 b	na	na	na	na	na
WP 4-3-3	na	na	na	na^3^	na	na	na
WP 4-4-2	5.1 d	18.2 bcd	na	13.1 b	na	na	na
WP 4-4-6	8.6 cd	9.7 c	na	na	na	na	0.0 e
WP 4-5-3	9.9 c	15.0 bcd	na	na	na	na	na
WP 4-10-4	na	na	na	na^3^	na	na	na
WP40	18.3 a	23.4 ab	23.6 a	16.1 b	8.0 c	20.3 a	24.6 ab
WP41	17.1 ab	23.5 ab	24.8 a	16.1 b	8.8 bc	20.8 a	25.7 a
WP42	14.0 b	21.0 ab	17.1 b	14.1 b	3.8 d	20.1 a	22.3 b
WW7	na	9.6 c	na	na	11.0 b	na	13.9 cd
PD1	na	na	11.3 c	na	18.3 a	na	16.7 c
2-79	14.5 ab	23.0 ab	14.3 bc	14.3 b	11.4 b	8.3 b	13.8 cd
Q8r1	10.0 c	18.5 bc	16.7 b	23.1 a	20.0 a	7.5 b	12.8 de

1 *Inhibition index = [y/(x + y)](100), for which x is the distance from the center of the fungal plug to the leading edge of the non-inhibited fungus, and y is the distance from the center of the bacterial colony to the inhibited edge of the fungus. Values are the averages of two experiments (n = 8). na, no activity. Letters indicate significant (P < 0.05) differences among means within each column, based on Fisher's LSD (Statistix, Tallahassee, FL)*.

2 *Variances between three experiments were unequal based on Bartlett's test; a representative experiment is shown (n = 4)*.

3 *No activity on full-strength PDA but partial activity on ¼X PDA*.

### Rapid assays to detect cyclic lipopeptides and volatile inhibitory compounds

Cyclic lipopeptide surfactant production was detected in *R. aquatilis* 4-3-1 and *P. fluorescens* 2-79 (positive control) in drop collapse assays. Droplets of whole-cell cultures and non-diluted culture supernatants, but not 1:10 supernatant dilutions, exhibited pronounced spreading and flattening on Parafilm, evidence of surfactant activity.

Volatile inhibitor activity was tested for endophyte strains in which the leading edge of the *R. solani* AG-8 colony usually did not reach the Petri plate perimeter relative to the no-endophyte control in inhibition index studies. Small but significant (*P* < 0.05) decreases in pathogen colony radii were observed for endophyte strains 4-2-2, 4-4-6, and WP40 but not for 4-5-3 or WP41 (Table 4). Reference strains, *P. protegens* Pf-5 and *Pseudomonas fluorescens* 2-79 that harbored loci for hydrogen cyanide (HCN) production, and endophyte 4-10-4 inhibited *R. solani* AG-8 in only one of the two experiments, indicating the transient and variable nature of the volatile activity. As expected, strain WP41 was negative. It was not known if strain Q8r1-96 would produce inhibitory amounts of HCN in our assay; it also was negative. There was no correlation between inhibition in the I plate and dual plate assays. For instance, strains 4-4-6 and 4-10-4 were active in the I plate assays but not in dual plate assays, and vice versa for strains 4-3-2 and WP41. Our findings suggest that multiple mechanisms of inhibition are involved in suppressing *R. solani* AG-8 *in vitro*.

**Table 4:** **Evidence for production of volatile inhibitory compounds in bacterial endophytes**.

**Strain^1^**	**Medium^2^**	**Colony radius (cm)^3^**	
		**Expt 1**	**Expt 2**
PDA control	PDA	34.4 a	20.2 a
WP 4-2-2	PDA	33.7 bcd	Not tested
WP 4-4-6	PDA	33.2 de	19.3 bc
WP 4-5-3	PDA	34.0 abc	20.0 a
WP 4-10-4	PDA	34.2 abc	19.0 c
WP40	PDA	33.0 e	19.3 bc
WP41	PDA	34.2 ab	19.8 ab
*Pf* Q8r1-96	PDA	34.0 abc	19.7 ab
*Pf* -5	PDA	33.5 cde	20.0 a
MG/L control	MG/L	34.7 a	31.3 a
WP 4-3-2	MG/L	34.2 a	30.8 a
*Pf* 2-79	MG/L	34.3 a	29.8 b

1 *Controls were done with medium only; WP41 was included as a negative endophyte control*.

2 *Preferred medium for rapid growth*.

3 *Radius of the R. solani AG8 colony 5 d and 4 d after growth on I-plates for experiments 1 and 2, respectively. n = 3. Letters indicate significant (P < 0.05) differences among means within each column, based on Fisher's LSD (Statistix, Tallahassee, FL)*.

### Comparative analysis of genomic regions related to the synthesis of anti-*Rhizoctonia* metabolites in *Burkholderia* species (WP40 and WP42)

**Figure 2:**
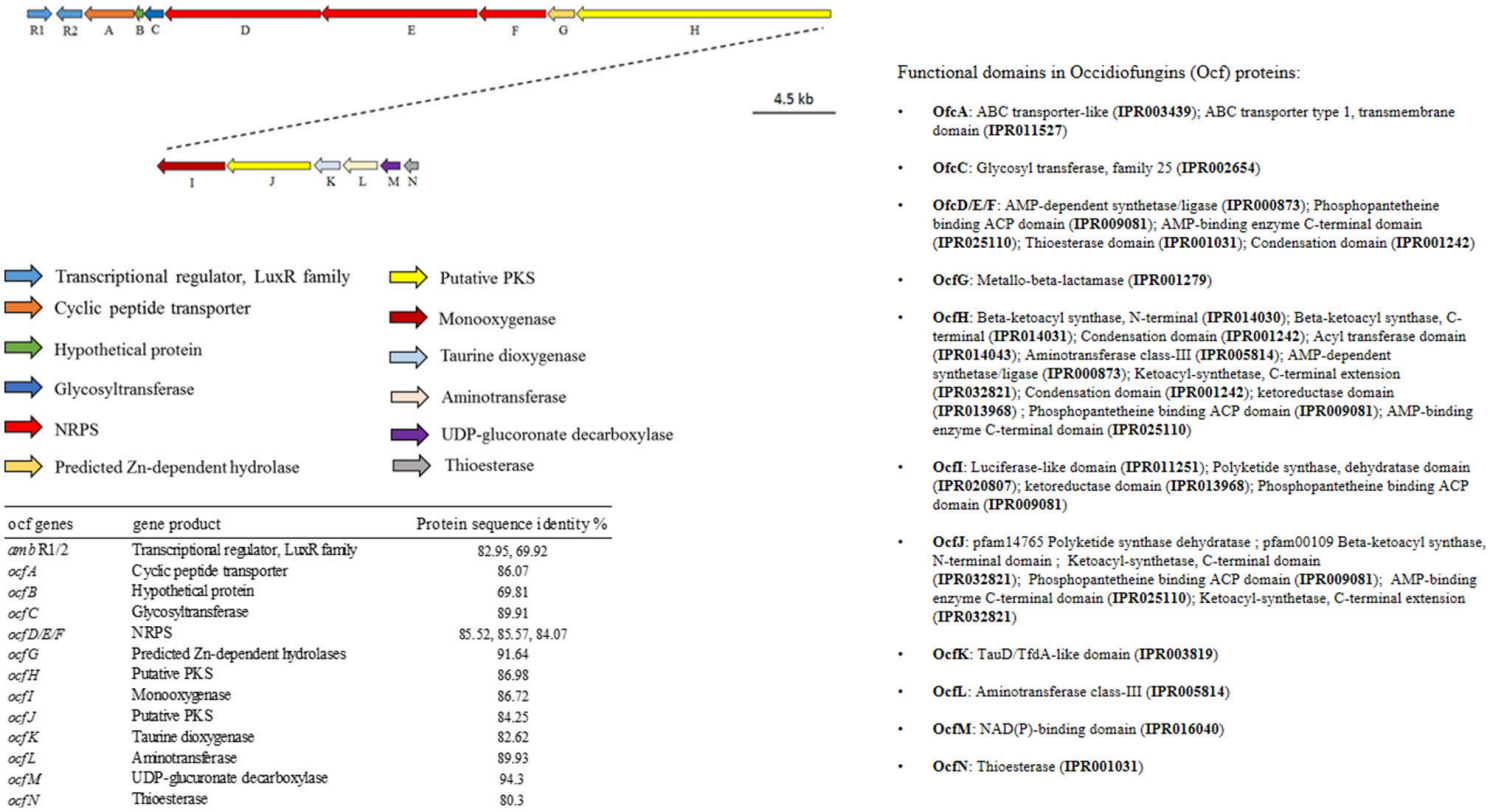
**Annotation and graphical representation of the *ocf* gene cluster in WP42 [Locus tags: EX20DRAFT_04106 (*amb*R1)–04091 (*ocf*N)] and WP40 [Locus tags: Ga0008008_11691 (*amb*R1)–116106 (*ocf*N)]**. The same colors were used for open reading frames (arrow) encoding for proteins with the same function (arrows). The right pane shows the protein domain.

**Figure 3:**
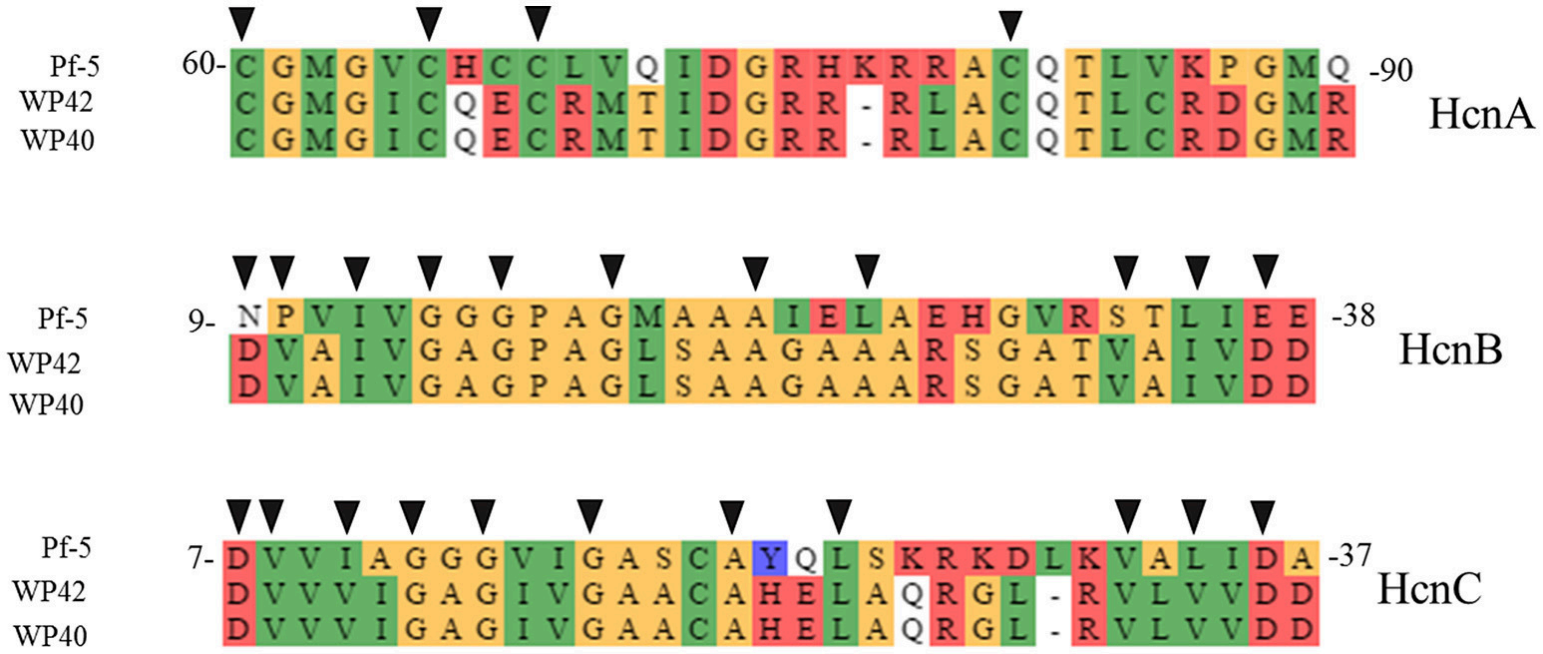
**Protein alignment of putative *hcn* biosynthetic genes in WP40 (Locus tag: Ga0008008_101290–101292), and WP42 (Locus Tag: EX20DRAFT_00138–00140) with HcnA, B, and C from *P. fluorescens* Pf-5**. The conserved amino acid residues of Fe-S binding sites, in HcnA, and ADP-binding motif, in HcnB and HcnC, important for hydrogen cyanide synthases function (Laville et al., 1998; Ryall et al., 2008), are indicated with black triangles.

Among the endophytic strains used in this study, only the genomes of WP40 and WP42 had been sequenced previously. These were analyzed to assess the presence of known gene clusters involved in the synthesis of secondary metabolites with antifungal activities. A syntenic block for the 56-kb *ocf* gene cluster of *B. contaminans* MS14, a known antifungal strain was detected in the genomes of WP40 and WP42 (Figure 2). As reported in Gu et al. (2009a), the *ocf* gene cluster consists of 15 open reading frames (*orf*) involved in the biosynthesis and secretion of *occidiofungin*, a cyclic glycopeptide, which has been reported to inhibit the growth of *R. solani, F. culmorum, P. ultimum* and other plant and human pathogens (Lu et al., 2009). The *ocf* region was annotated in antiSMASH as hybrid NRPS-Type-1 PKS gene cluster that contains three *orf* each of which encodes for a non-ribosomal peptide synthetase (NRPS), and a coding sequence for a Type 1 PKS. However, gene clusters such as pyrrolnitrin (*prn*ABCD), HQSs (*hmq*ABCDEFG), AFC-BC11 and PKSs genomic island, involved in the synthesis of known antifungal compounds, are absent in WP40 and WP42. Moreover, a putative *hcnABC* gene cluster involved in the synthesis of HCN was also detected in the WP40 and WP42 genomes (Figure 3). The synthesis of HCN seems to be a common feature of plant growth promoting *Burkholderia* strains. Furthermore, genome annotation of PD1 revealed the presence of chitinase and secreted protein annotated as chitin deacetylase, which may play a role to inhibit the fungal growth.

### IAA production

**Figure 4:**
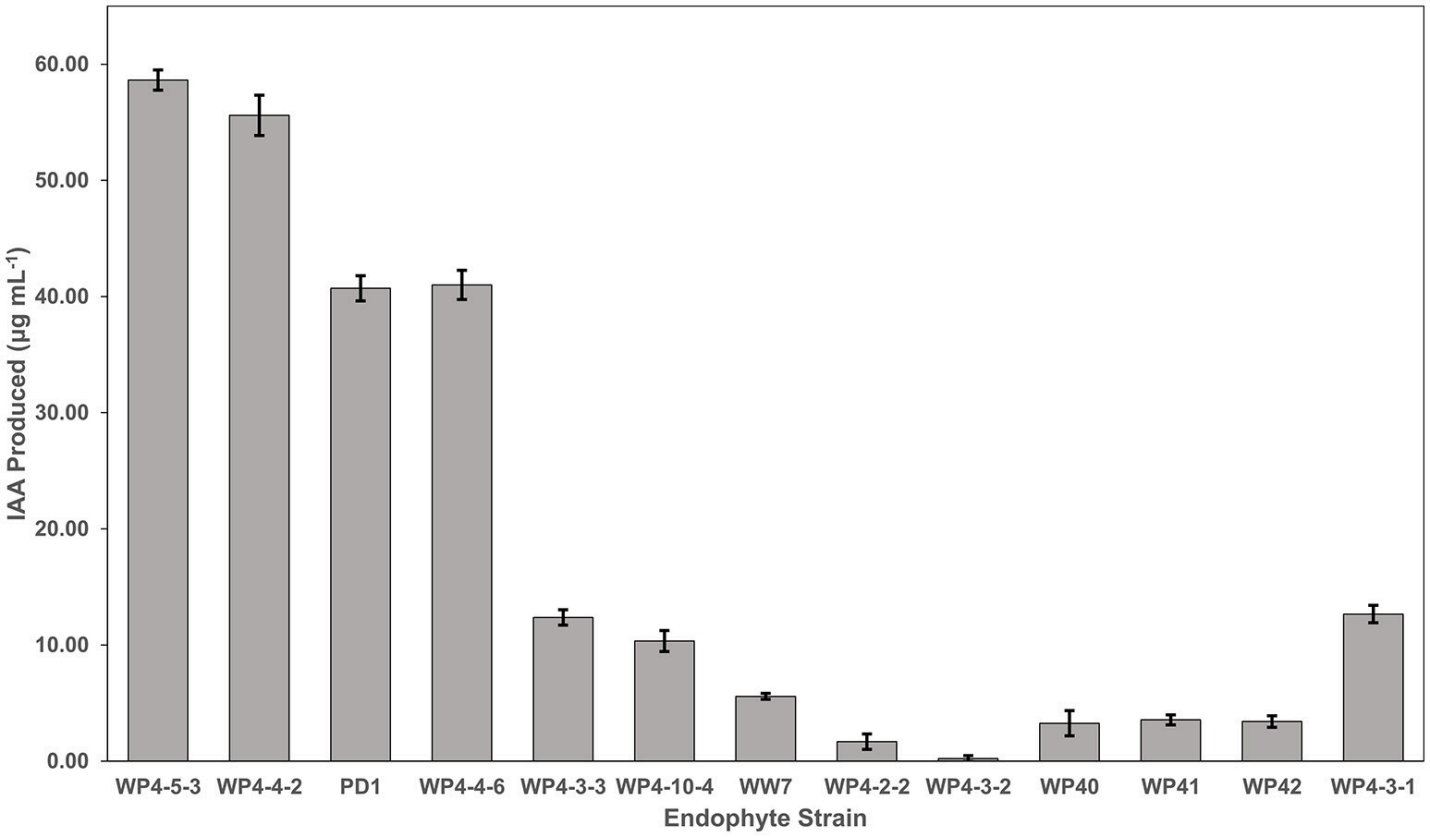
**IAA production by the Salicaceae endophytes**. Strains were grown in triplicate for 4 days in TYC broth containing 0.1% L-tryptophan. The IAA amount produced by each endophyte strain was calculated using the standard curve.

The amount of IAA produced by the different endophytes when supplemented with L-tryptophan varied between the different strains, generally along species lines (Figure 4). The two strains producing the highest auxin levels were both *Rahnella aquatilis* strains (WP4-5-3 and WP4-4-2). The next two highest producers were both *Pseudomonas* species (WP4-4-6 and PD1). The two *Curtobacterium* strains (WP4-4-2 and WP4-10-4) and the yeast strain (WP4-3-1) produced moderate levels of the phytohormone. All five of the *Burkholderia* strains produced the least amount of the auxin. A negligible amount (<1.30 μg ml-1 of endophyte culture) of IAA was observed for all the endophyte strains without addition of L-tryptophan (data not shown).

### Phosphate solubilization

**Figure 5:**
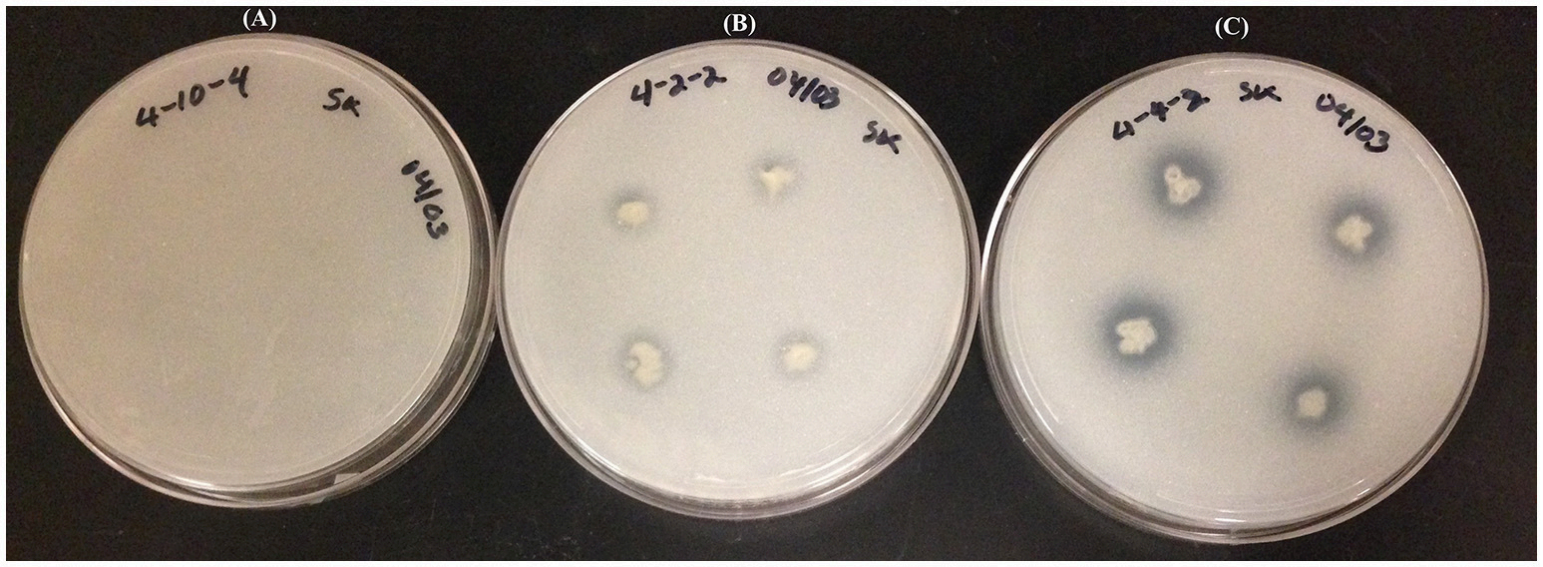
**Tricalcium phosphate (NBRIP) plates showing the phosphate solubilization gradient: Plate (A);** no solubilization, Plate **(B)**; moderate solubilization, and Plate **(C)**; high solubilization.

Tricalcium phosphate/NBRIP plates were used for the phosphate solubilization assay. This assay was done once but strains were tested in quadruplicate positions on individual petri plates. The halo area surrounded by endophyte colonies was a zone of phosphate solubilization, which was assessed by calculating PSI as described earlier (Khan et al., 2015). Several strains solubilized tricalcium phosphate more effectively than others (Figure 5). The best performing strains were WP40, WP41, WP42, WP 4-4-2, WP 4-5-3, and scored more than 1.5 PSI. Some strains; WP 4-2-2, WP 4-3-2, WP 4-4-6, PD1, and WW7 showed a relatively inconspicuous halo area. No solubilization was observed by strains WP 4-3-1, WP 4-3-3, and WP 4-10-4.

### Siderophore production

**Figure 6:**
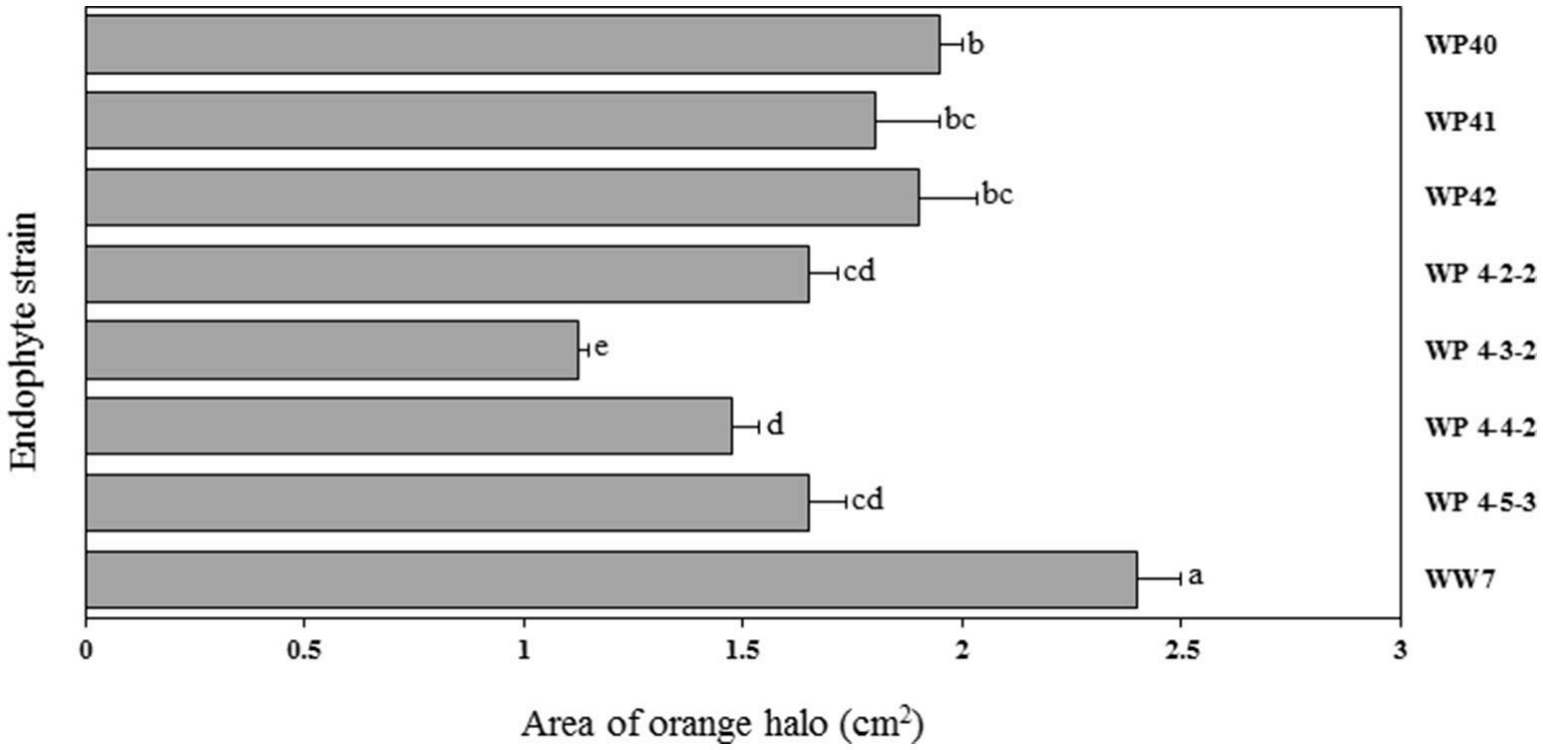
**Area of orange halo (cm^2^) displayed by different Salicaceae endophytes in CAS agar plates**. Histograms that do not share a letter are significantly different at <0.05 probability level.

CAS agar plates were used to observe the siderophore activity of Salicaceae endophytes. The orange halo area surrounded by endophyte colonies was measured to assess the siderophore production *in-vitro*. Poplar endophytes; WP40, WP41, WP42, WP 4-2-2, WP 4-3-2, WP 4-4-2 and WP 4-5-3, and willow endophyte WW7 showed siderophore activity creating the orange halo area contiguous with colony growth (Figure 6). No siderophore activity was observed by strains, WP 4-3-1, WP 4-4-6, WP 4-10-4, and PD1. The largest orange halo area; 7.539 cm^2^ was observed in willow endophyte, WW7.

### Plant growth promoting features in poplar *Burkholderia* (WP40 and WP42) genomes

**Figure 7:**
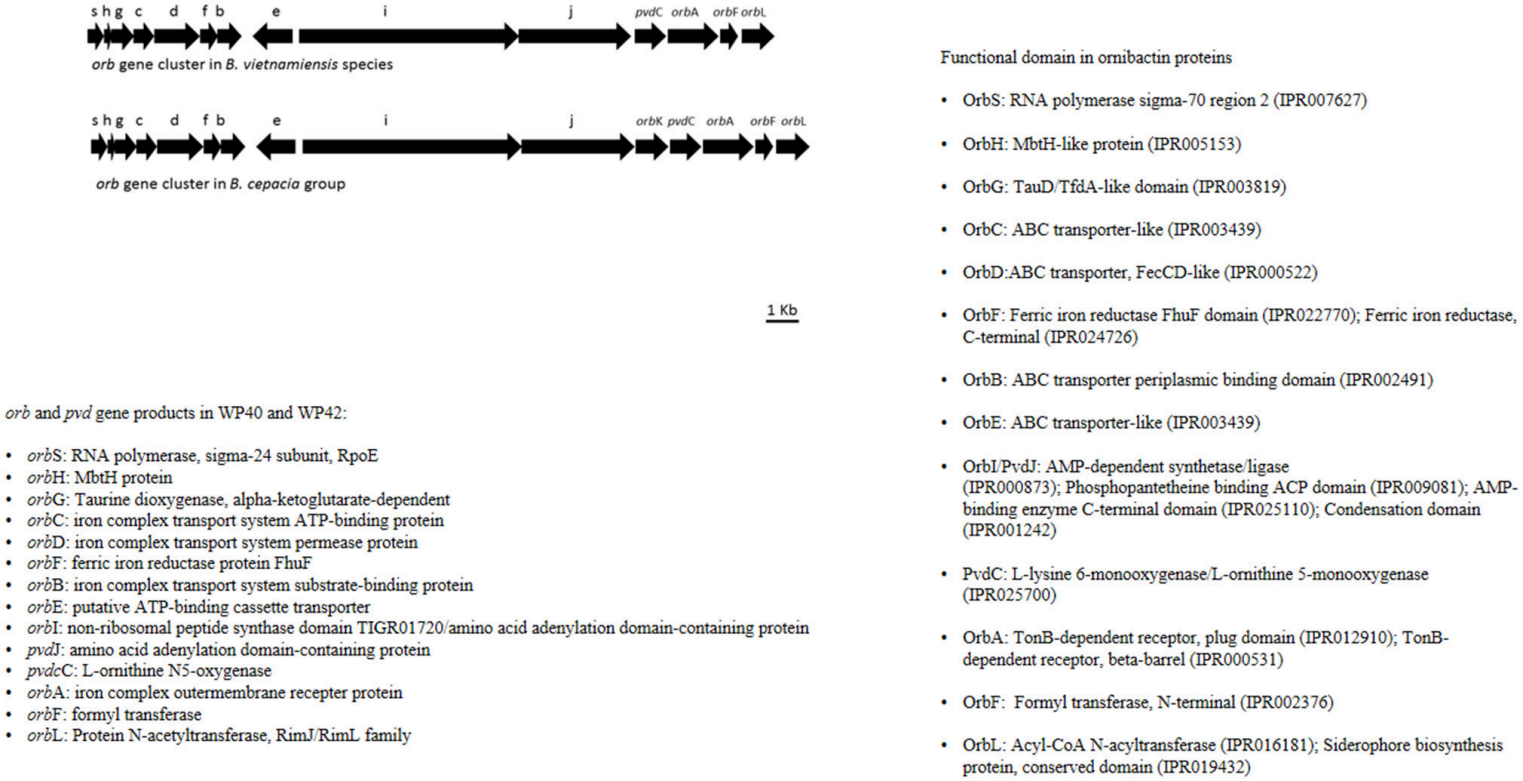
**Annotation of *orb* and *pvd* genes in WP42 [Locus tags: EX20DRAFT_02566(*orb*S)–02579 (*orb*L)], and WP40 [Locus tags: Ga0008008_110136 (*orb*S)–110149 (*orb*L)], and *B*. *cepacia* groups**.

Plant associated (endophytic or rhizospheric) *Burkholderia* species have promising plant growth promoting properties (BNF, phosphate solubilization, siderophore production, degradation of aromatic compounds, and phytohormone production). From a genome analysis of poplar endophytic *Burkholderia* strains WP40 and WP42, it was revealed that they carry putative genes that are responsible for the above mentioned characteristics related to plant growth promotion. As observed for other *Burkholderia* strains, the *nifHDK* operon was detected in WP40 and WP42 along with 1-aminocyclopropane-1-carboxylate (ACC) deaminase coding sequence. In addition, these strains, WP40 and WP42, have the pyrroloquinoline quinone (pqqBCDE) operon that is essential to solubilize rock phosphates in soil. An interesting feature of WP40 and WP42 is the presence of a non-canonical ornibactin (*orb*) gene cluster, which encodes for biosynthesis of siderophore compounds. Compared to other *orb* gene clusters, this non-canonical cluster is present in all core genes but lacks N-acetyltransferase coding sequence orbK, which is not essential in the synthesis of ornibactins (Franke et al., 2014). Interestingly, this gene arrangement has never been observed and seems to be unique in the *Burkholderia* species (Figure 7).

### Acetylene reduction assay

**Figure 8:**
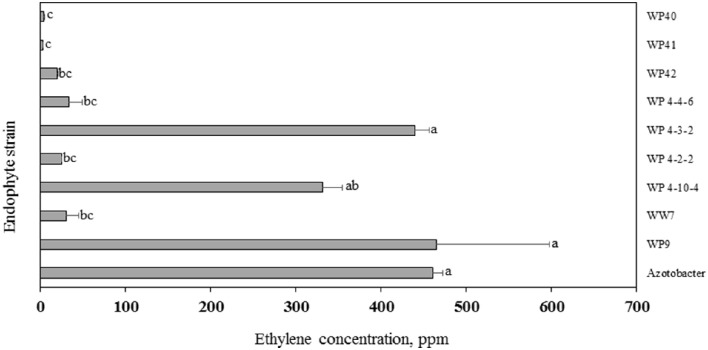
**Acetylene reduction assay**. Ethylene produced by endophyte strains after 24 h of exposure to acetylene. Histograms that do not share a letter are significantly different at <0.05 probability level.

In the ARA, the activity of the nitrogenase enzyme leads to reduction of acetylene gas to ethylene, which is monitored through gas chromatography. The amount of ethylene provides an estimate of N fixed by diazotrophs. More than 300 ppm concentration of ethylene was produced by strains WP9, WP 4-3-2, WP 4-10-4, WP 4-4-6, and positive control *Azotobacter* sp. (Figure 8). A relatively smaller amount of ethylene was produced by endophyte strains WP40, WP41, WP42, WP 4-4-2, and WW7.

### Analysis of the nitrogenase subunit gene, *nifH*

Endophyte strains WP40, WP41, WP42, WP 4-4-2, and WP 4-5-3 were found positive to universal *nifH*, and *nifH* primers. Strains WP 4-4-2 and WP 4-5-3 were positive to *nifH-b1*.

## Discussion

In this study, we characterized the bio-control potential of Salicaceae endophytes including other microbial properties, which are associated with plant growth promotion. We used *in-vitro* microbiological techniques to observe the phenotypic functionality of these endophytes related to plant growth promotion and suppression of different plant pathogens, and genome analyses to understand the possible underlying mechanisms for these functions. The main objective of this study was to investigate whether any of the poplar or willow endophytes have antagonistic activities over the growth of soil borne plant pathogens including *R. solani* AG-8, *F. culmorum, Ggt*, and *P. ultimum*. Previous studies suggested that endophytes are vital for poplar and willow plants to colonize the flood prone areas of the Snoqualmie River, which are largely dominated by sand and rock gravels (Doty et al., 2005, 2009, 2016). With the large number of available strains from this environment, it is important to focus the research on those with the greatest potential of providing plant health benefits. No previous studies tested for antagonistic activities of these endophytes on plant pathogens. Among the 55 poplar and 4 willow endophyte strains used in the 2005 to 2016 studies, ten poplar endophytes and one willow endophyte used here showed antagonistic activities against *R. solani* AG-8, *F. culmorum, Ggt*, and *P. ultimum*. These strains were then further tested for additional growth promoting activities including IAA production, phosphate solubilization, siderophore production, and BNF.

Irrespective of the different types of media, all endophyte strains grew robustly. *Pseudomonas* species produced distinct yellow pigments in MG/L. The media containing glutamate or mannitol are known to enhance yellow pigment production in *Pseudomonas* (Osawa et al., 1963). The yellow pigment has the potential to chelate the iron from the environment, which is a required nutrient for bacterial growth (Meyer and Abdallah, 1978). The dual plate assays displayed variable antagonistic responses against *F. culmorum, Ggt, P. ultimum*, and *R. solani* AG-8. However, the endophyte *Burkholderia* spp. (WP40, WP41, and WP42) had antagonistic activities across all four tested pathogens. More importantly, the antagonistic performance of these strains was equally as impressive as that of the positive control *Pseuodomonas* strains. It was demonstrated that *Burkholderia* produced a glycolipopeptide named occidiofungin, which has an inhibitory effect on various fungal pathogens (Gu et al., 2009a,b). From a genome analysis of WP40 and WP42, a gene cluster responsible for biosynthesis of an *ocf* -like cyclic peptide was discovered. Among various genes embedded within that 56-kb *ocf* gene cluster, ambR1 was reported as a key regulatory gene controlling the biosynthesis of occidiofungin (Gu et al., 2009b), which is also conserved in the genomes of poplar *Burkholderia*.

Liquid chromatography-mass spectrometry and nuclear magnetic resonance spectroscopy of poplar endophytes WP40 and WP41 were conducted to analyze for the anti-fungal compounds, especially occidiofungin. However, the peaks that correspond to occidiofungin mass were not detected in the spectrum originated from WP40 and WP41 (data not shown). The anti-fungal compounds might not be synthesized to detectable levels when grown under medium away from the fungus. The strain WP 4-3-1 showed the evidence of CPL synthesis but did not show any antagonistic effect on dual plate assays. Other strains that showed antagonistic effects were negative for CLP secretion assay but showed fungal growth suppression in volatile inhibitory and dual plate assays. From the genome analysis, the *hcnABC* locus that encodes for VOCs like HCN was detected in the genomes of WP40 and WP42, which corroborates the results of dual plate assays and the volatile inhibitory assay. Despite an evident inhibitory effect in fungal growth, studies regarding the effectiveness of *Burkholderia* strains to produce discrete amounts of HCN are dissonant (Ryall et al., 2008; Gilchrist et al., 2013). Thus, it is a possibility that the strong anti-fungal activities observed in WP40 and WP42 resulted from the combination of synthesizing volatile compounds like HCN, and glycopeptides like occidofungin. The antagonistic activities of bacterial VOCs against phytopathogenic fungi were discussed elsewhere (Weisskopf, 2013). Further functional analysis of putative anti-fungal genes would provide the necessary information about the mechanisms used by endophytes to arrest the growth of various plant pathogens, which would be also useful to formulate biofungicides.

The antagonistic properties of fungal endophytes of poplar have been investigated before (Busby et al., 2016); however, to our knowledge, our study is the first to analyze these properties of bacterial endophytes recovered from poplar and willow plants. In the past, studies were concentrated on soil bacteria or rhizospheric bacteria as a means of bio-control agents (Beneduzi et al., 2012; Santoyo et al., 2012). In contrast, the endophytic lifestyle of microorganisms could offer systemic tolerance against many plant pathogens as they colonize the entire plant body and stably persist. In addition, they use internal plant tissues for nutrition and multiplication, which excludes them from competition with other microbes present in the phyllosphere or rhizosphere. In our study, the bio-control potential of Salicaceae endophytes was not restricted only to *Burkholderia* species but also observed in *Pseudomonas, Rahnella*, and *Curtobacterium* indicating that poplar endophyte consortia have the potential to inhibit the growth of several plant pathogens. From earlier studies, it is concluded that the poor performance of bio-control in field conditions is due to poor root colonization by bio-control agents (Compant et al., 2005). The endophyte strains used in our study were isolated from poplar and willow branches. These endophytes can colonize both roots and shoots of a variety of plant species and may provide promising results in the field conditions to manage different soil pathogens. Previous studies have shown that these endophytes were competent to colonize the endosphere of rice and conifer seedlings (Kandel et al., 2015; Khan et al., 2015).

Some concerns have been raised about the use of *Burkholderia* strains in agriculture since some strains are associated with the *Burkholderia cepacia* complex (Bcc), opportunistic pathogens in cystic fibrosis (CF) patients (Chiarini et al., 2006). However, members of this genera are wide-spread in the environment including within plants (Perin et al., 2006; Compant et al., 2008). Interestingly, pathogenicity tests performed on *B. vietnameiensis* strains isolated from environmental niches were negative for pathogenicity (Bernier et al., 2003; Agnoli et al., 2012). On the other hand, most strains isolated from CF patients show a strong or moderate pathogenic behavior, suggesting that specific genetic traits might be unique and positively selected in those strains isolated from CF patients (Saini et al., 1999; Chu et al., 2002; Cieri et al., 2002). This indicates that a phylogenetic classification does not fully represent the pathogenic potential of a strain (Eberl and Vandamme, 2016) and that more studies aimed to understand the molecular effectors exploited by opportunistic pathogens of the Bcc complex should determined. Angus et al. (2014) reported that plant symbiotic *Burkholderia* species lack virulence-associated loci required in mammalian pathogenesis. We used comparative based genomic analysis to distinguish the secretion systems of poplar *Burkholderia* strains from known pathogenic *B*. strains. Key pathogenesis-related gene clusters encoding for components of the Type III, Type IV (Sajjan et al., 2008), Type VI-5 (Schwarz et al., 2010) and Type VI-1 (Burtnick et al., 2011) secretion systems were absent from the genomes of WP40 and WP42 (data not shown) in contrast to pathogenic *Burkholderia* strains, suggesting that the secretion systems of poplar *Burkholderia* strains are most likely deficient to initiate infection, and thus might be separated from pathogenic Bcc strains.

All the endophyte strains that were tested for IAA biosynthesis were positive in the presence of L-tryptophan, with the exception of WP4-3-2. We were unable to detect any notable amount of IAA in the absence of L-tryptophan. It is possible that the colorimetric method used in this study to quantify IAA may not be sensitive enough to detect low levels of IAA biosynthesis. However, similar findings were reported for endophytes isolated from a variety of other plant species (Shi et al., 2009; Xin et al., 2009a; Videira et al., 2012). L-tryptophan is commonly available in plant exudates (Kamilova et al., 2006; Hardoim et al., 2008). The requirement for exogenous precursor may be a reflection of the mutually beneficial interactions of the symbiont and the host, with the microbe converting a host metabolite to a growth promoting substance for the host (Kravchenko et al., 2004; Kamilova et al., 2006; Xin et al., 2009b). The poplar endophyte strains varied in the amount of auxin produced, with the *Rahnella* and *Pseudomonas* strains producing the highest levels and the *Burkholderia* strains producing the least. Another poplar endophyte, *Burkholderia* strain WPB, not included in this study, produced similar levels of the auxin, and stimulated root growth by 72% (Xin et al., 2009b); therefore, even low levels produced under these *in vitro* conditions can have *in vivo* a strong impact on the plant. The *Rhodotorula graminis* yeast strain, WP4-3-1, produced a moderate level of auxin. Other endophytic yeast strains of poplar have been reported to produce this phytohormone (Xin et al., 2009a). IAA synthesis produced by endophytes can stimulate root growth as well as may influence other developmental processes such as apical dominance, tropic responses, flowering, and fruiting (Spaepen and Vanderleyden, 2011; Spaepen, 2015). Since the endophytes were capable of IAA production even though they colonized aboveground tissue, this may support the hypothesis that endophyte-produced auxins may have these impacts in addition to increasing rooting. However, the locations of IAA production *in planta* by these strains have yet to be determined.

The majority of the endophyte strains having antagonistic activities also solubilized phosphorus in NBRIP medium containing insoluble phosphorus. Many past studies reported that endophytic or growth promoting soil bacteria solubilize inaccessible soil phosphorus into bioavailable forms, providing potential phosphorus resources for plants to uptake for their growth and development (Gamalero and Glick, 2011; Oteino et al., 2015). Furthermore, evidence of siderophores production was observed in many endophyte strains. Siderophore are iron chelating organic compounds produced by microbes or plants to accrue iron from environments. Plant growth promotion and relief of iron deficiency symptoms have been demonstrated through microbial siderophores in different crop plants (Ahmed and Holmström, 2014; Saha et al., 2015).

Endophyte strains WP40, WP41, WP42, WP 4-4-2, and WP 4-5-3 reduced acetylene into ethylene in the ARA, indicating N-fixation, and were found positive for the *nifH* gene by PCR. However, strains WP 4-3-2, WP 4-10-4, and WP 4-4-6 were ARA positive but none of these had a *nifH* PCR product. Although the primers are considered as “universal,” it is often the case that this primer set does not amplify *nifH* of diazotrophic strains (Bürgmann et al., 2004). Previous studies also reported the discrepancy in relationship between the *nifH* profiles and ARA results (Deslippe et al., 2005; Patra et al., 2006). Future studies for these strains would be to have the genomes sequenced. Furthermore, strains 4-4-2 and 4-5-3 were *nifH* positive but ARA negative. The *in vitro* culture conditions for ARA may not be favorable for all strains to show N-fixing activity. Nitrogenase activities would differ with the culture medium, growth conditions, and growth stages of bacteria (Lin et al., 2012) or maybe it is only active *in planta*.

The results from genome analyses corroborate the phenotypic observations for BNF, phosphate solubilization, IAA production, and siderophore production. Since we observed the *nifHDK* gene cluster encoding for the nitrogenase enzyme in WP40 and WP42, we can postulate that they can fix atmospheric N, which is potentially available to the host plants to assimilate. The ARA results of WP40 and WP42 also supports the hypothesis of BNF. In addition, the *pqq* operon involved in phosphate solubilization, and tryptophan-2-monooxygenase and ACC deaminase for phytohormone production/modulation were observed in the WP40 and WP42 genomes. It has been shown that the expression of *pqqABCDE* genes stimulates gluconic acid production, which solubilizes the insoluble phosphates (Richardson et al., 2009; Oteino et al., 2015). In addition, it is claimed that ACC deaminase producing bacteria help plants to mitigate the biotic and abiotic stresses by lowering the ethylene level in plants that would otherwise interfere with the plant physiological processes and eventually damage the entire plant body (Glick, 2014).

The utilization of microbial symbionts in crop cultivation offers an ecologically sound and sustainable way of farming. The results from *in vitro* assays suggest that Salicaceae endophytes have the potential to increase plant growth, possibly through the mechanisms of N-fixation, IAA production, phosphate solubilization, and siderophore production but may also protect the host plants from pathogen-induced plant diseases. Recent advances in molecular biology and functional genomics will help to expand the existing understanding of plant-endophyte interactions. Integration of multiple microbial strains as a single consortium can offer different benefits to the crop plants. Further investigations about outcomes of plant-endophyte interactions in different ecological settings and under field conditions would be beneficial to utilize the full plant growth promoting potential of endophytes.

## Author contributions

Conceived and designed the experiments: SLK, SD, and PO. Performed the experiments: SLK, PMJ, NL, and KM. Analyzed the data: SLK, PMJ, AF, NL, KM, PO, GM, AH, SHK, and SD. Contributed reagents/materials/analysis tools: SLK, SD, PO, and AF. Wrote the paper: SLK, AF, PO, PMJ, and SD.

## Funding

This study was supported by a grant from the USDA NIFA grant 2012-68002-19824, and USDA Project Number 2090 22000 016 00D and Washington Grain Commission grant 3061-4548 (PO).

### Conflict of interest statement

The authors declare that the research was conducted in the absence of any commercial or financial relationships that could be construed as a potential conflict of interest.
