# Corrigendum: An *In vitro* Study of Bio-Control and Plant Growth Promotion Potential of Salicaceae Endophytes

**DOI:** 10.3389/fmicb.2018.02067

**Published:** 2018-09-05

**Authors:** Shyam L. Kandel, Andrea Firrincieli, Pierre M. Joubert, Patricia A. Okubara, Natalie D. Leston, Kendra M. McGeorge, Giuseppe S. Mugnozza, Antoine Harfouche, Soo-Hyung Kim, Sharon L. Doty

**Affiliations:** ^1^School of Environmental and Forest Sciences, College of the Environment, University of Washington, Seattle, WA, United States; ^2^Department for Innovation in Biological, Agro-Food and Forest Systems, University of Tuscia, Viterbo, Italy; ^3^Department of Biology, University of Washington, Seattle, WA, United States; ^4^Wheat Health, Genetics and Quality Research Unit, USDA-ARS, Pullman, WA, United States; ^5^Department of Plant Pathology, Washington State University, Pullman, WA, United States

**Keywords:** bio-control, Salicaceae endophytes, soil borne plant pathogens, *Burkholderia*

In the original article, there was a mistake in Table 1 as published. The correct GenBank accession number of strains; WP 4-3-1, WP 4-4-2, WP 4-4-6, WP 4-5-3, and WP42 are KU500895, KU500894, KU500891, KU500892, and KF597276, respectively. The correct identity of strains WP 4-4-2 and WP 4-5-3 is *Rahnella* sp. The accession number of strains; WP 4-3-1, WP 4-4-2, WP 4-4-6, WP 4-5-3, and WP42 were misprinted. The identity of strains WP 4-4-2 and WP 4-5-3 was listed as *Rahnella aquatilis*. The corrected Table 1 appears below.

**Table 1:** Endophyte strains used in this experiment.

**Strain**	**Identity**	**Origin**	**Appearance**	**PGPA^a^**	**GenBank accession no**.	**rRNA sequence size, partial (bp)**
WP 4-2-2	*Burkholderia* sp.	Poplar root	Buff	IAA, NA	KU495920	903
WP 4-3-1	*Rhodotorula graminis*	Poplar stem	Pink	CLP, IAA	KU500895	554
WP 4-3-2	*Burkholderia* sp.	Poplar stem	Buff, mucoid	Sd, NA	KU500893	947
WP 4-3-3	*Curtobacterium* sp.	Poplar stem	Bright yellow	IAA	KU550576	962
WP 4-4-2	*Rahnella* sp.	Poplar stem	Buff, mucoid	IAA, Sd	KU500894	966
WP 4-4-6	*Pseudomonas* sp.	Poplar stem	Pale yellow	IAA, NA	KU500891	1,032
WP 4-5-3	*Rahnella* sp.	Poplar stem	Buff, mucoid	Sd, IAA	KU500892	1,076
WP 4-10-4	*Curtobacterium* sp.	Poplar leaf	Bright yellow	NA, IAA	KU550577	859
WP40	*Burkholderia* sp.	Poplar stem	Buff	*ofn, orb, hcn*, Sd, NA, IAA	KF597274	1,494
WP41	*Burkholderia* sp.	Poplar stem	Buff	*ofn, orb*, Sd, NA, IAA	KF597275	1,494
WP42	*Burkholderia* sp.	Poplar stem	Buff	*ofn, orb*, Sd, *hcn*, NA, IAA	KF597276	1,494
WW7	*Curtobacterium* sp.	Willow stem	Pale yellow	IAA, Sd, NA	KU523564	926
PD1	*Pseudomonas putida*	Poplar stem	Buff	IAA	KF443801	1,496
*Pf* 2-79	*P. fluorescens*	Soil	Bright yellow	CLP, *phz*		
*Pf* Q8r1-96	*P. brassicacearum*	Soil	Buff	CLP, *dapg, plt, prn*		

a *Plant growth promoting activities: cyclic lipopetide (CLP) determined using the drop collapse assay; indole acetic acid (IAA) determined on YEM medium plus L-trpytophan using Salkowski reagent; gene clusters for the antifungal metabolites occidiofungin (ofn), ornibactin (orb), hydrogen cyanide (hcn), diacetylphloroglucinol (dapg), phenazine (phz), pyoluteorin (plt) and pyrrolnitrin (prn) loci determined using PCR; siderophore (Sd) determined by orange halo on CAS medium, nitrogenase activity (NA) determined by acetylene reduction assay*.

The authors apologize for this error and state that this does not change the scientific conclusions of the article in any way.

The original article has been updated.

## Conflict of interest statement

The authors declare that the research was conducted in the absence of any commercial or financial relationships that could be construed as a potential conflict of interest.

