# Supplementary material for: An In vitro Study of Bio-Control and Plant Growth Promotion Potential of Salicaceae Endophytes
Source: Front Microbiol. 2017 Mar 13;8:386. doi: 10.3389/fmicb.2017.00386 (PMC5347143; doi:10.3389/fmicb.2017.00386)
Supplement: Supplementary file 1 [file Table1.docx]

**Table S1 Poplar and willow endophytes used to test anti-fungal activities.**

| Strain names | Closest 16s rDNA match |
| --- | --- |
| PTD1 | *Rhizobium tropici* |
| WPB | *Burkholderia vietnamiensis* |
| WP1 | *Rhodotorula graminis* |
| WP5 | *Rahnella* species |
| WP9 | *Burkholderia* species |
| WP19 | *Acinetobacte*r *calcoaceticus* |
| Snoq 117.2 | *Pseudomonas* species |
| WP40 | *Burkholderia* species |
| WP41 | *Burkholderia* species |
| WP42 | *Burkholderia* species |
| WW17 | *Burkholderia vietnamiensis* |
| WW5 | *Sphingomonas* species |
| WW6 | *Pseudomonas putida* |
| WW7 | *Curtobacterium* species |
| WP 4-1-1 | Unidentified |
| WP 4-1-2 | Unidentified |
| WP 4-1-3 | Unidentified |
| WP 4-1-4 | Unidentified |
| WP 4-2-1 | Unidentified |
| WP 4-2-2 | *Burkholderia* sp. |
| WP 4-2-3 | Unidentified |
| WP 4-3-1 | *Rhodotorula graminis* |
| WP 4-3-2 | *Burkholderia* sp*.* |
| WP 4-3-3 | *Curtobacterium* sp. |
| WP 4-3-4 | Unidentified |
| WP 4-3-5 | Unidentified |
| WP 4-3-6 | Unidentified |
| WP 4-3-7 | Unidentified |
| WP 4-3-8 | Unidentified |
| WP 4-4-1 | Unidentified |
| WP 4-4-2 | *Rahnella aquatilis* |
| WP 4-4-3 | Unidentified |
| WP 4-4-4 | Unidentified |
| WP 4-4-5 | Unidentified |
| WP 4-4-6 | *Pseudomonas* sp. |
| WP 4-5-1 | Unidentified |
| WP 4-5-2 | Unidentified |
| WP 4-5-3 | *Rahnella aquatilis* |
| WP 4-6-1 | Unidentified |
| WP 4-6-2 | Unidentified |
| Strain | Closest 16s rDNA match |
| WP 4-6-3 | Unidentified |
| WP 4-6-4 | Unidentified |
| WP 4-7-1 | Unidentified |
| WP 4-7-2 | Unidentified |
| WP 4-7-3 | Unidentified |
| WP 4-7-4 | Unidentified |
| WP 4-8-1 | Unidentified |
| WP 4-8-2 | Unidentified |
| WP 4-8-3 | Unidentified |
| WP 4-8-4 | Unidentified |
| WP 4-8-5 | Unidentified |
| WP 4-8-6 | Unidentified |
| WP 4-9-1 | Unidentified |
| WP 4-9-2 | Unidentified |
| WP 4-9-3 | Unidentified |
| WP 4-9-4 | Unidentified |
| WP 4-10-1 | Unidentified |
| WP 4-10-2 | Unidentified |
| WP 4-10-3 | Unidentified |
| WP 4-10-4 | *Curtobacterium* sp. |
| WP 4-10-5 | Unidentified |
